# 

**Published:** 1855-08

**Authors:** 


*To Subscribers.*—We again enclose receipts to those of our subscribers who have paid during the past month, with the exception of those in Michigan and Wisconsin, who hold the receipts of Mr. Jesse Clement, and those in New York, who have the receipt of Mr. D. C. Stevens. As these gentlemen are our authorized agents, our own acknowledgme nt is unnecessary. Mr. Clement will next visit Southern Michigan, Indiana, Illinois, and Iowa. Mr. Stevens will probably visit the southern tier of counties in this state. We are sorry to state that our June edition is exhausted. We can, however, supply the July No. to some extent, and are printing a larger edition for August. The portrait of Dr. Flint will, however, be sent to all new subscribers.

---

*To Correspondents.*—Communications from Dr. Isidor Glück, Prof. Hamilton, Dr. J. L. Chandler, and Dr. Wm. J. Meacham, are received, and shall have place in our next, if possible.

